# Low morbidity and mortality with COVID‐19 in sickle cell disease: A single center experience

**DOI:** 10.1002/jha2.87

**Published:** 2020-08-30

**Authors:** Preethi Ramachandran, Abhilash Perisetti, Balachandar Kathirvelu, Mahesh Gajendran, Snigdha Ghanta, Ifeanyichkwu Onukogu, Ted Lao, Faiz Anwer

**Affiliations:** ^1^ Department of Hematology and Oncology Brookdale University Hospital and Medical Center Brooklyn New York; ^2^ Division of Gastroenterology and Hepatology University of Arkansas for Medical Sciences Little Rock Arkansas; ^3^ Department of Rehabilitation Sciences The University of Texas at El Paso El Paso Texas; ^4^ Department of Internal Medicine Texas Tech University Health Sciences Center El Paso Paul L Foster School of Medicine El Paso Texas; ^5^ Department of Internal Medicine Brookdale University Hospital and Medical Center Brooklyn New York; ^6^ Department of Hematology/Oncology Stem Cell Transplantation Cleveland Clinic Cleveland Ohio

**Keywords:** anemia, general hematology, hematological oncology, hemoglobin disorders, sickle cell disease

## Abstract

Coronavirus disease 2019 (COVID‐19) is caused by SARS‐CoV‐2 infection, which evolved into a global pandemic within a short time. Individuals with sickle cell disease (SCD) suffer from underlying cardiopulmonary comorbidities and are at risk of severe complications such as pneumonia, acute chest syndrome, thrombosis, stroke, and multiorgan failure. Whether COVID‐19 poses a high risk of morbidity and mortality in SCD patients remains unclear. Patients with SCD and COVID‐19 can present with overlapping clinical features such as respiratory symptoms with ground‐glass infiltrates, hyperinflammatory state, and increased risk of thromboembolism. This highlights the need to maintain a low threshold for testing for COVID‐19 infection among symptomatic and hospitalized SCD patients. We report a case series of nine hospitalized SCD patients diagnosed with COVID‐19 from March 18, 2020 to April 30, 2020 at a tertiary medical center in New York City. The mean age of the study population was 27.9 years, and interval since onset of symptoms and hospital presentation was 1–2 weeks. All patients in our series improved and were discharged home. This limited study shows that SCD patients, who are perceived to be high risk, maybe somehow protected from severe symptoms and complications of COVID‐19 infection.

## 1 INTRODUCTION

Novel coronavirus disease (COVID‐19) is caused by severe acute respiratory syndrome corona virus‐2 (SARS‐CoV‐2) [1]. There have been more than 5 million cases worldwide with 355,942 deaths as of May 27, 2020 [2]. COVID‐19 predominately targets the respiratory system causing acute respiratory distress syndrome (ARDS), which remains the major cause of morbidity and mortality in COVID‐19. SARS‐CoV‐2 can also affect the gastrointestinal tract, and patients can present with altered taste, abdominal pain, and diarrhea [1, 3, 4]. Patients with COVID‐19 can present with severe cytokine release syndrome (cytokine storm) affecting different organ systems [5]. Sickle cell disease (SCD) patients are at risk of developing severe complications if affected by a viral illness. It is unclear, if COVID‐19 can cause serious complications in SCD patients, and if SCD itself or its complications can be a risk factor for severe COVID‐19 disease. In this case series, we describe individuals with a history of SCD hospitalized due to COVID‐19, their clinical presentation, and outcomes.

## 2 METHODS

COVID‐19 diagnosed adults (18 years or older) with a history of SCD (homozygous hemoglobin S [HbSS], compound heterozygous HbS and HbC [HbSC]) were identified from Brookdale University Health System between March 18 and April 30, 2020. The local institutional review board approved the study protocol and granted a waiver of informed consent due to its retrospective nature. The clinical characteristics, laboratory, and outcomes data were extracted from electronic medical records in a standardized report form. A total of 725 patients who tested positive for SARS‐CoV‐2 by reverse transcription‐polymerase chain reaction in nasopharyngeal swab or sputum samples were screened, and 9 patients with SCD were identified. Quantitative data were shown as the mean ± standard deviation (SD) or as a percentage. Qualitative variables were expressed as absolute and relative frequencies.

## 3 RESULTS

Data from nine hospitalized SCD patients among 725 diagnosed with symptomatic COVID‐19 were reported in this study. The demographic and clinical characteristics of these patients are shown in Table 1. The main symptoms at presentation were fever (77.8%), myalgia (66.7%), cough and back pain (33.3%) followed by less common symptoms including dyspnea, gastrointestinal symptoms, and sore throat. Preceding history of complications of SCD including iron overload (44.4%), acute chest syndrome (33.3%), pulmonary hypertension (22.2%), and avascular necrosis (22.2%) was reported among these patients. History of asthma was reported in 44.4%. Table 2.

**TABLE 1:** Demographics, clinical features, laboratory values, and outcomes

Characteristic	Mean	SD	Lab findings	*n*	%
Age	27.9	7.2	Anemia	9	100
BMI	25.6	4.7	Elevated bilirubin	8	88.9
	n	%	Elevated ferritin	7	77.8
Female	4	44.4	Leukocytosis	5	55.6
Race			Elevated AST	4	44.4
African American	9	100	Elevated ALT	1	11.1
Symptoms			Blood group		
Fever	7	77.8	A	5	55.5
Myalgia	6	66.7	O	4	44.4
Cough	3	33.3	Sickle cell disease	Mean	SD
Back pain	4	44.4	Hgb A1	9.4	10.2
Dyspnea	1	11.1	Hgb A2	2.9	1.1
GI symptoms	2	22.2	Hgb F	11.0	8.5
Sore Throat	1	11.1	Hgb S	72.8	13.3
Prev Complications due to SCD		Hgb C	5.0	15.0	
H/o transfusion	8	88.9	Home Medication	n	%
Ferritin > 1000	4	44.4	Folic acid	8	88.9
Acute chest	3	33.3	Hydrea	6	66.7
Avascular necrosis	2	22.2	Opiates	8	88.9
Pulm HTN	2	22.2	Others	0	0
Pulmonary embolism	1	11.1	Outcomes	Mean	SD
Priapism	1	11.1	Length of stay	7.1	1.9
CVA	1	11.1		*n*	%
Comorbities			Survival rate	9	100
Asthma	4	44.4	Shock	1	11.1
Smoker	1	11.1	Ventilation	0	0

Abbreviations: ALT, alanine aminotransferase; AST, aspartate aminotransferase; CVA, cerebrovascular accidents; Hgb, hemoglobin; SD, standard deviation.

**TABLE 2:** Clinical characteristics, comorbidities, and complications in COVID‐19 SCD patients

Case	Age	Gender	Sickle cell Type	Symptoms at presentation	Comorbidities	Duration of Symptoms	Previous SCD complications	Past 1 year admission
#1	27	M	HBSS	Cough, fever, nausea, fatigue, myalgia, back pain	Asthma	1 week	Ferritin > 1000, H/o transfusion	19
#2	28	F	HBSS	Back pain and headache	None	1 week	Acute chest, pulmonary HT (TRJV > 2.5), H/o transfusion	11
#3	21	M	HBSS	Cough, fever, sore throat, nausea, fatigue, myalgia	Schizophrenia	1 week	Priapism, H/o transfusion	1
#4	21	M	HBSS	Fever, myalgia, back pain	None	5 days	Acute chest, ferritin > 1000, avascular necrosis, H/o transfusion	NA
#5	31	F	hbsc	Cough, fever, myalgia, dysgeusia, Rt hip pain, chest pain, loss of appetite	Asthma	2 days	Pulmonary embolism	2
#6	37	M	HBSS	Fever, fatigue, myalgia, generalized weakness	None	2 weeks	Microalbuminuria, vaso‐occlusive episodes	2
#7	40	M	HBSS	Fever	CVA, seizure, DVT	1 day	Acute chest, CVA, pulmonary HT (TRJV > 2.5), ferritin > 1000, H/o transfusion (chronic)	2
#8	19	F	HBSS	Fever, LOW BACK PAIN, AND LEG pain	Asthma	2 weeks	H/o transfusion	16
#9	27	F	HBSS	Myalgia	Chronic Ulcers, Asthma	1 week	Ferritin > 1000, avascular necrosis H/o transfusion	3

Abbreviations: CVA, cerebrovascular accident; DVT, deep vein thrombosis; HT, hypertension; NA, not available; TRJV, tricuspid regurgitant jet velocity.

**FIGURE 1:**
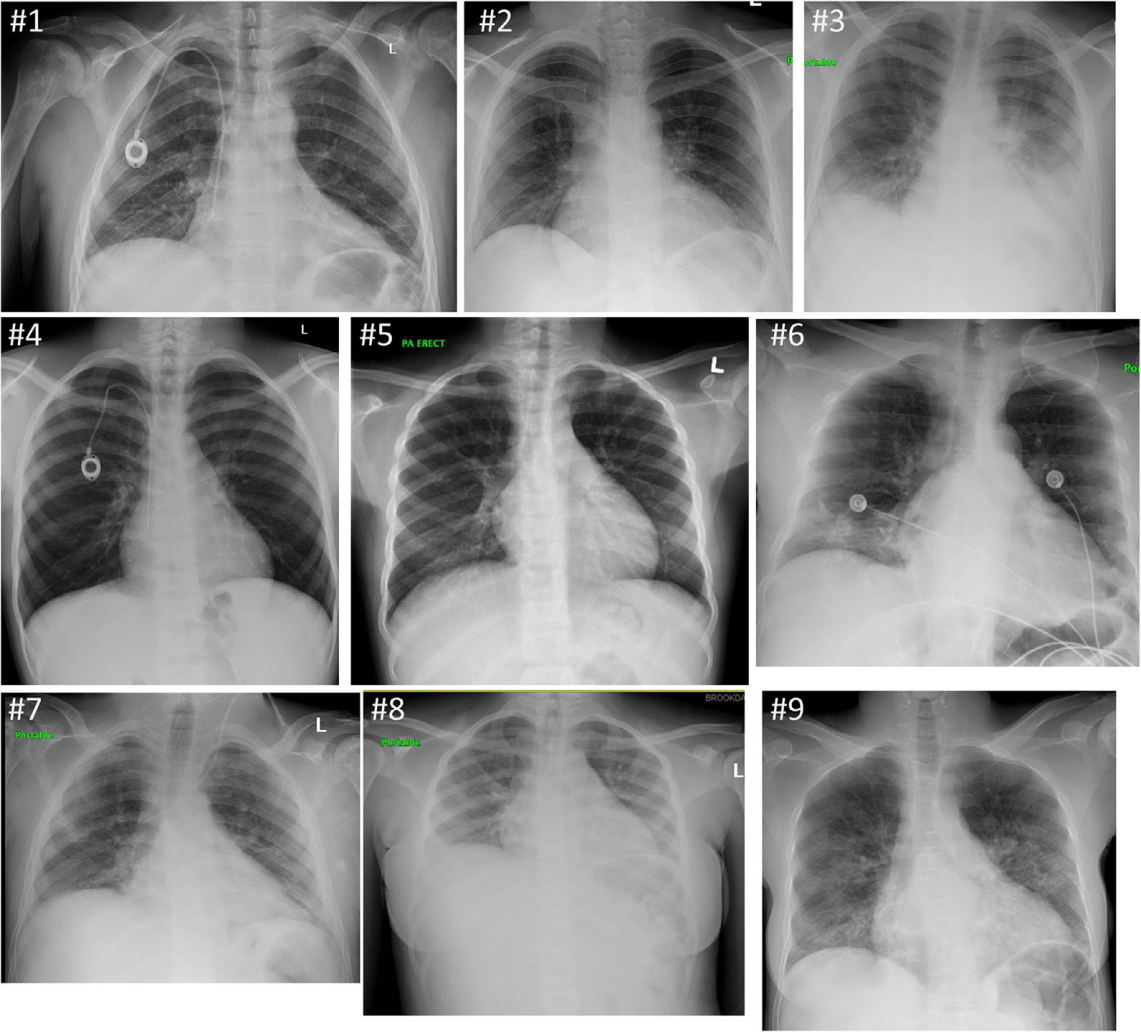
Chest radiograph of COVID‐19 patients with SCD

Laboratory data showed anemia (100%) with mean hemoglobin (post) of 7.8 g/dL (SD 1.1), elevated bilirubin (88.9%), elevated ferritin (77.8%), and leukocytosis (55.6%). Laboratory values at baseline and during hospitalization are summarized in Table 3. Home medications included folic acid (88.9%), opiate analgesics (88.9%), and hydroxyurea (66.7%). None of the patients were on l‐glutamine, voxeletor, or crizanlizumab. All HBSS patients had HBS ranging from 49.1% ‐ 92.5% and Fetal Hemoglobin (HBF) ranging from 1.5% to 30.4%. Around 50% of patients were treated with hydroxychloroquine and azithromycin. None of the patients received antiviral therapy or IL‐6 inhibitor. About 66% of the patients needed simple blood transfusion support during their hospital admission as summarized in Table 4. Vital signs, radiological features, management, and outcomes are noted in Table 4. The duration of symptoms before presentation ranged between 1 and 2 weeks. All patients except one showed respiratory parenchymal changes that ranged from subtle hazy appearance to frank infiltrates (Figure 1). All of them had sickle cell crisis and received hydration and analgesics.

**TABLE 3:** Laboratory data in COVID‐19 SCD patients

	Hb		WBC		ALC		ANC		Creatinine		PT		PTT		Fibrinogen		D‐dimer		ALT		AST		Bilirubin		Lactate		CRP		Ferritin		Troponin		LDH	
Case	Pre	Post	Pre	Post	Pre	Post	Pre	Post	Pre	Post	Pre	Post	Pre	Post	Pre	Post	Pre	Post	Pre	Post	Pre	Post	Pre	Post	Pre	Post	Pre	Post	Pre	Post	Pre	Post	Pre	Post
#1	8.3	8.0	9.1	9.8	0.5	0.45	6	5.6	0.5	0.45	1.3	1.43	40	36.5	NA	NA	1072	NA	24	31	27	84	0.2	7.9	1.7	NA	1.5	7.6	NA	1210	0.012	0.012	605	1881
#2	8.0	6.3	12	8.7	0.6	0.74	7.6	5.9	0.6	0.74	1.4	NA	25	NA	623	NA	800	NA	19	18	34	29	6.2	3.5	0.8	NA	NA	NA	900	NA	0.012	NA	768	637
#3	10.0	9.6	3.5	8.2	0.6	0.55	1.9	5.3	0.6	0.55	1.2	NA	31	NA	NA	NA	150	NA	12	45	21	37	2.3	2.1	NA	NA	8.8	NA	135	968	0.012	0	666	NA
#4	6.8	6.7	9	16	0.5	0.57	2.7	7.3	0.5	0.57	1.3	1.2	35	40.3	NA	NA	500	NA	22	35	30	29	3.6	3.2	0.9	NA	5.8	NA	550	857	0.012	NA	900	361
#5	9.0	8.6	13	13	0.6	0.71	9.8	NA	0.6	0.71	1.2	NA	26	NA	700	NA	NA	243	27	17	28	25	1.1	1.4	NA	0.8	NA	NA	22	NA	0.012	0.012	917	NA
#6	10.0	8.1	8.1	5.3	0.94	3.74	3.5	41.6	0.94	3.74	1.18	1.93	26.3	30.4	NA	1102	NA	44514	26	80	52	64	3.9	1.9	NA	6.7	NA	18	NA	974	0.012	0.317	NA	1673
#7	8.0	5.8	5.6	6.5	0.5	0.74	4.2	4.7	0.5	0.74	1.2	NA	35	NA	NA	NA	800	NA	30	100	36	100	0.9	0.5	2.1	0.9	NA	NA	5000	3830	0.012	0	442	757
#8	10.0	7.6	9.9	29	0.5	0.53	4.2	19.6	0.5	0.53	1.2	1.26	25	22.1	NA	NA	NA	NA	18	51	32	70	1.9	3.7	0.8	1.7	3.8	6.4	137	1340	0.012	NA	788	1215
#9	9.0	8.0	11	24	0.5	0.6	6	16.5	0.5	0.6	1.1	1.27	32	28.1	NA	488	NA	529	17	24	31	49	2.7	2.7	1.1	NA	NA	3.9	NA	5000	0.012	0.012	788	1096

Pre: Baseline/preadmission; Post: average values during hospitalization. Abbreviations: ALC, absolute lymphocyte count; ALT, alanine aminotransferase; ANC, absolute neutrophil count; AST, aspartate aminotransferase; CRP, C‐reactive protein; LDH, lactate dehydrogenase; NA, not available; Pt, prothrombin time; PTT, partial thromboplastin time; WBC, white blood cell count.

**TABLE 4:** Radiographic findings and management in COVID‐19 SCD patients

				COVID medication Azi Plaq									
Case	Age	Gender	Home SCD medications	Azi	Plaq	Other medication	Blood transfusion	Respiratory rate/min	O_2_ nadir	Radiological finding	Respiratory support	ICU admission	Outcome
#1	27	M	Folic acid, hydrea, opiates	No	No	None	0	20	95%	Subtle hazy appearance to lower lungs	Nasal cannula	No	Discharged
#2	28	F	Folic acid, opiates	Yes	No	Doxycycline	1 (Day 5)	20	95%	Subtle hazy appearance to lower lungs	No	No	Discharged
#3	21	M	Folic acid, hydrea, opiates	Yes	Yes	Ceftriaxone	1 (Day 3)	40	85%	Left lower lung infiltrates	HFNC	No	Discharged
#4	21	M	Folic acid, hydrea, opiates	No	Yes	Ceftriaxone, doxycycline	0	20	95%	Perihilar interstitial opacities	Nasal CANNULA	No	Discharged
#5	31	F	Folic acid, opiates	Yes	Yes	None	0	19	95%	Mild left basilar hazy opacity, right basilar opacity	No	No	Discharged
#6	37	M	None	No	Yes	Cefepime, vancomycin	4 (Day 2 and 4)	32	88%	Bibasilar infiltrates	NIPPV	Yes	Discharged
#7	40	M	Folic acid, hydrea, opiates	No	No	None	3 (Day 1 and 7)	21	91%	Consolidation in both lungs, most pronounced in both lower lung fields	Nasal cannula	No	Discharged
#8	19	F	Folic acid, hydrea, opiates	Yes	No	Clindamycin, doxycycline	2 (Day 3)	18	98%	Clear lungs	No	No	Discharged
#9	27	F	Folic acid, hydrea, opiates	NA	NA	Ceftriaxone, vancomycin	3 (Day 1 and 3)	18	94%	Extensive bilateral diffuse ground glass opacities particularly in the lung bases	No	No	Discharged

Abbreviations: Azi, azithromycin; HFNC, high flow nasal cannula; NA, not available; NIPPV, nasal intermittent positive pressure ventilation; Plaq, plaquenil.

Age matched controls were compared with SCD for clinical outcomes. Fifty‐three out of 725 were among those aged 18–40 years. Of these, 19 patients needed ICU admission with four needing intubation. Four died with mortality of 5.6%. Among the nine SCD patients, only one needed ICU admission without the need for intubation. No deaths were observed, and all were discharged. The average length of hospital stay was slightly longer in SCD (7.1 days) than the age matched control (6.8 days) as summarized in Table 5.

**TABLE 5:** Comparison of SCD and age‐matched patients on O_2_ saturation, intubation, length of stay, and mortality

	Sickle cell disease	Age matched (18‐40 years)
No of patients	9 of 725	53 of 725
ICU admission	1	19
mean O_2_ saturation	92.9%	97.3%
Intubation	0	4
Length of stay	0‐16 days (mn 7.1)	0‐28 days (mn 6.8)
Mortality	0 (0%)	3 (5.6%)

## 4 DISCUSSION

Patients with SCD are a unique subset of hematological disease population who are postulated to be at higher risk of developing multiple and severe complications with COVID‐19 due to multiple organ derangement due to SCD complications. Data on clinical manifestations of SARS‐CoV‐2 in SCD is scarce. SCD patients at baseline have anemia, increased risk of infections, and vaso‐occlusive crisis (VOC). Acute respiratory illnesses, in general, are a major cause of mortality and morbidity in SCD due to increased risk of developing pneumonia, pulmonary VOC disease, and acute chest syndrome [6]. Infections are major causes of morbidity and mortality in SCD individuals due to tissue hypoperfusion, functional hyposplenism, disproportionately high inflammatory overload, or hypoventilation [7, 8]. Furthermore, viral infections such as H1N1, seasonal influenza, Zika can present with increased virulence in these individuals [9, 10, 11]. If SARS‐CoV‐2 viral infection produces such hyperinflammatory response in SCD individuals is yet to be reported. The interplay between symptoms or complications of SARS‐CoV‐2 in patients with SCD who has anemia at baseline, varying levels of hemoglobin variants such as Hb S, Hb F, iron overload, current or recent exposure to hydroxyurea, other prescription drugs, and underlying lung pathology remains unknown.

## 5 PRESENTING SYMPTOMS AND COMPLICATIONS

Reported ARDS incidence with COVID‐19 is around 15‐33%, but the data on the severity of lung involvement, acute chest syndrome (ACS), ARDS, and other presenting symptoms with COVID‐19 in individuals with SCD is limited [12]. Nur et al. reported two patients who presented with ACS, severe back pain, and extremity pain. One patient recovered completely; however, the clinical course for the second individual is not known [13]. In a case series, McCloskey et al. reported 10 SCD patients from the United Kingdom with favorable outcomes except for one patient for whom morbidity and mortality were attributed to multiple underlying comorbidities [14]. Similarly, Hussain et al. reported four patients with SCD who had favorable outcomes. VOC, musculoskeletal symptoms dominated in these patients with one patient showed predominant gastrointestinal symptoms of nausea, vomiting, and diarrhea [15]. Gastrointestinal (GI) symptoms could be a dominant presenting symptom in 10–15% of COVID‐19 cases, rarely patients present with GI only symptoms, without respiratory or hematological manifestations [3, 4].

## 6 OVERLAP IN PATHOPHYSIOLOGY AND COMPLICATIONS

Presenting features of COVID‐19 and SCD complications without SARS‐CoV‐2 infection can overlap significantly and a high level of vigilance is needed while providing care to patients with SCD especially during the pandemic period [13]. SARS‐CoV‐2 binds to angiotensin‐converting enzyme‐2 (ACE‐2) expressed on multiple tissues predominantly in the oral cavity (tongue), lung, heart, kidney, and ileal enterocytes, making them vulnerable to viral injury [16]. ACE‐2 activity is also noted in the lymphocytes in the lungs and digestive tract; however, its clinical importance is unclear [16]. COVID‐19 patients develop hyperinflammatory response (cytokine storm), which can lead to higher vascular permeability, extensive microthrombi formation leading to multiorgan failure, and death [9]. Similarly, SCD patients can develop multiple pathophysiological changes due to vaso‐occlusion, which include inflammation (with increased sickling), hemolysis (with increased adhesion to endothelium), hypoxia, and ischemic reperfusion injury (by reactive oxygen species) [17, 18]. Due to the history of multiple transfusions, SCD patients have high ferritin levels, a finding common in severe COVID‐19 patients where it serves as a marker of inflammation [19]. It is conceivable that if both pathologies coexist in a patient, an enhanced inflammatory cascade is expected. However, such changes have not been reported and have not resulted in less favorable outcomes among SCD patients with COVID‐19.

## 7 WHAT AND HOW

It remains to be studied how some of these pathophysiological pathways potentially interact to mitigate adverse effects of COVID‐19. SCD mostly affects the African American race [20]. The same ethnicity showed a strong association with severe COVID‐19 disease symptoms [21]. It is unclear if severe COVID‐19 presentation highlights the social disparities at play or the high underlying comorbidity burden which is present in this population. However, this case series and recently published literature review suggest that outcomes are not necessarily worse for SCD patients. All reported cases had anemia, and around 80% of our patients had predominantly HBS hemoglobin (range: 49.1‐92.5) with higher HBF (range: 1.5‐30.4), use of opiates, folic acid, and hydroxyurea was documented in 60–80% of these cases. It is unclear whether hemoglobin S or Hemoglobin F or prescription medication use in these patients provided any protective effect from severe complications of COVID‐19 in this hospitalized population.

## 8 LIMITATIONS

Limitations of our data include the retrospective nature of our data captured in this case series. An alternative explanation of the low risk of complications includes the younger age of patients with SCD, low incidence of HTN, and their low comorbidity disease burden. To further explore this issue of complications with COVID‐19, preclinical models and larger clinical studies with more data or registry studies need to be conducted and updated frequently [22]. Such studies can provide knowledge about preventative strategies and risk mitigation for COVID‐19 patients. Targeted screening of SCD patients for COVID‐19 symptoms is needed to identify the disease at the early stages for prompt monitoring and intervention.

## CONFLICT OF INTEREST

The authors declare no conflict of interest.

## AUTHOR CONTRIBUTIONS

Conceptualizations, methodology, Supervision: PR, FA; manuscript writing, reviewing, and editing: PR, BK, FA; statistical and data analysis: AP, MG; data collection &literature review: SG, IO, TL, AP.
